# Dissemination and Implementation Science Approaches for Occupational Safety and Health Research: Implications for Advancing Total Worker Health

**DOI:** 10.3390/ijerph182111050

**Published:** 2021-10-21

**Authors:** Rebecca J. Guerin, Samantha M. Harden, Borsika A. Rabin, Diane S. Rohlman, Thomas R. Cunningham, Megan R. TePoel, Megan Parish, Russell E. Glasgow

**Affiliations:** 1 Division of Science Integration, Centers for Disease Control and Prevention, National Institute for Occupational Safety and Health, 1090 Tusculum Ave., MS C-10, Cincinnati, OH 45226, USA; hul6@cdc.gov; 2 Department of Human Nutrition, Foods, and Exercise, Virginia Tech, Blacksburg, VA 24060, USA; harden.samantha@vt.edu; 3 Herbert Wertheim School of Public Health and Human Longevity Science, University of California San Diego, La Jolla, CA 92037, USA; barabin@health.ucsd.edu; 4 UC San Diego Altman Clinical and Translational Research Institute, Dissemination and Implementation Science Center, University of California San Diego, La Jolla, CA 92037, USA; 5 Department of Family Medicine and Dissemination and Implementation Science Program of Adult and Child Center for Outcomes Research and Delivery Science, University of Colorado Anschutz Medical Campus, Aurora, CO 80045, USA; russell.glasgow@cuanschutz.edu; 6 Occupational and Environmental Health, University of Iowa, 145 N. Riverside Drive, Iowa City, IA 52245, USA; diane-rohlman@uiowa.edu (D.S.R.); megan-tepoel@uiowa.edu (M.R.T.); 7 Oregon Institute of Occupational Health Sciences, Oregon Health & Science University, Portland, OR 97239, USA; megan.parish@confluencehealth.org

**Keywords:** dissemination and implementation science, Total Worker Health, translational science, occupational safety and health, evidence-based interventions, health equity

## Abstract

Total Worker Health^®^ (TWH), an initiative of the U.S. National Institute for Occupational Safety and Health, is defined as policies, programs, and practices that integrate protection from work-related health and safety hazards by promoting efforts that advance worker well-being. Interventions that apply the TWH paradigm improve workplace health more rapidly than wellness programs alone. Evidence of the barriers and facilitators to the adoption, implementation, and long-term maintenance of TWH programs is limited. Dissemination and implementation (D&I) science, the study of methods and strategies for bridging the gap between public health research and practice, can help address these system-, setting-, and worker-level factors to increase the uptake, impact, and sustainment of TWH activities. The purpose of this paper is to draw upon a synthesis of existing D&I science literature to provide TWH researchers and practitioners with: (1) an overview of D&I science; (2) a plain language explanation of key concepts in D&I science; (3) a case study example of moving a TWH intervention down the research-to-practice pipeline; and (4) a discussion of future opportunities for conducting D&I science in complex and dynamic workplace settings to increase worker safety, health, and well-being.

## 1. Introduction

The Total Worker Health^®^ (TWH) approach from the National Institute for Occupational Safety and Health (NIOSH), part of the U.S. Centers for Disease Control and Prevention (CDC), first arose in 2003. TWH is defined as policies, programs, and practices that integrate protection from work-related safety and health hazards by promoting efforts to advance worker well-being [1,2]. Interventions with a TWH focus have been demonstrated to improve workplace health effectively and more rapidly than wellness programs alone [3,4]. For example, the Wellworks-2 intervention integrated an occupational safety and health (OSH) program targeted at reducing workplace exposure hazards with a health promotion (HP) program to reduce tobacco consumption and increase healthy eating [3,5]. Results from a randomized controlled trial of Wellworks-2 demonstrated, among a number of outcomes, significantly greater smoking quit rates and reduced hazardous substance exposure ratings than an HP-only program did [3,5]. Although TWH efforts and activities have increased in recent years and have generated both national and international attention and interest [2], TWH is still an emerging area with minimal research addressing interventions appropriate to the changing nature of work in the United States and worldwide. Complex OSH challenges—including large-scale public health crises such as the COVID-19 pandemic, the rise of globalization, automation, demographic shifts, increased psychosocial hazards (including high job demands, work-related fatigue, and job stress), and the interaction of work and nonwork factors [6]—require wider and faster adoption of TWH approaches that benefit workers, employers, and society [7].

To increase the impact (including representative reach to diverse worker populations), effectiveness, and integration (including the adoption, implementation, and maintenance [8,9]) of TWH activities, systematic approaches are needed to shed light on the complex processes involved in moving evidence-based TWH interventions into sustained practice [2,3,10,11,12]. Systematic and other reviews acknowledge the need for dissemination and implementation (D&I) science—defined as the study of methods and strategies for bridging the gap between public health research and practice [13]—to advance the TWH field. For example, Punnett and colleagues [12] suggest that D&I science can be utilized in TWH investigations to characterize factors affecting TWH program uptake, successful implementation, scale-up, and sustainment. Furthermore, Anger and colleagues [3] call for research in D&I science to “determine what works”, and what should therefore be included in future TWH research agendas. D&I science inquiry can be expanded not only to explain the important question of “what works”, but also to address key contextual issues such as what works, for whom, how, in what settings, and how it is sustained over time [14].

A helpful, plain language tool from Curran [15] to describe key, D&I concepts posits that:

- The intervention is THE THING;
- Effectiveness research looks at whether THE THING works;
- Implementation research looks at how best to help people (e.g., employers and workers)/(work)places DO THE THING;
- Implementation strategies are the stuff researchers do to try to help people/(work)places DO THE THING as designed/intended (i.e., with fidelity), such as provide training, technical assistance, and/or incentives;
- Main implementation outcomes are HOW MUCH and HOW WELL they DO THE THING.

Missing from the Curran definition is another critical consideration, and that is related to the context of the intervention. As mentioned above, context asks the question when, where, how, with whom, under what circumstances, and why does “the thing” work? [9,14,16,17]. Contextual factors identified in TWH studies include: (1) the legal–regulatory environment (e.g., state laws with respect to union representation); (2) employer characteristics, policies, or benefits (e.g., availability of health insurance coverage or paid sick leave); (3) work organization (e.g., shift work); and (4) social or economic factors (e.g., income or availability of community resources to support or promote health) [18]. Given the variety of contexts in which the TWH model can be implemented and studied—with variation in employers, work environments, and workers—understanding the factors that influence the effectiveness of integrated interventions is important. Currently, there is a gap in the TWH research in this area [10].

In sum, applications of D&I science hold promise for addressing major OSH challenges, [19,20] including those addressed through the TWH paradigm. More investment in resources tailored to meet the needs of TWH researchers are required to build capacity in D&I science theories, models, frameworks [21,22,23,24], designs, methods [25,26], and pragmatic measures [27,28] for conducting rigorous D&I studies.

The purpose of this paper is to draw upon the D&I literature to provide TWH researchers and practitioners with: (1) an overview of D&I science; (2) a plain language explanation of key concepts in D&I; (3) a case study example of moving a TWH intervention down the reseach-to-practice pipeline; and (4) future opportunites for D&I science in TWH. As more scientific knowledge has been generated to date about the methods that successfully promote implementation as compared to those that advance dissemination, the approaches discussed in this article are most relevant to the successful implementation of TWH programs. This paper fills several gaps in the TWH literature by providing an accessible overview of D&I science through a synthesis of key literature and proposing ideas for leveraging D&I to advance TWH in U.S. and global workpalces.

## 2. What Is D&I Science? A Brief Overview for TWH Researchers and Practitioners

Across many fields, the science of the systematic implementation of evidence-based interventions—broadly defined as the “7 Ps”: programs, practices, principles, procedures, products, pills, and policies [25]—lags behind the science of developing the interventions themselves [29]. Many overlapping factors related to the characteristics of interventions (e.g., high cost or poor fit with stakeholder needs), the settings where programs are implemented (e.g., resource constraints, lack of regulatory/policy support), and the characteristics of recipients (e.g., lack of buy-in for the program) lead to limited uptake, implementation, and sustained use of these programs [30,31]. In OSH specifically, this research-to-practice lag has substantial implications for the health, safety, and well-being of the global workforce [20].

D&I science is a growing field of study that examines the processes by which scientific evidence is adopted, implemented, and sustained in community or clinical settings [13,30]. Although a relatively new and transdisciplinary field of study, D&I has a strong historical foundation [30,31]. The field is concerned with changing systems by understanding context, leveraging an established evidence base, documenting outcomes, and characterizing the underlying mechanisms of change so that positive results can be replicated in other community-based and especially low-resource settings [30,32].

Other key characteristics and implications of D&I science, adapted from Glasgow and Chambers [33], with tailored considerations for TWH researchers are presented in Table 1.

Another useful concept from Brownson and colleagues [34] is referred to as designing for dissemination, implementation, and sustainment (D4DIS). D4DIS is a process to ensure that the products of research (such as new technologies, training, and health communication messages) are developed with the needs, resources, and time frames of the target audience in mind. It is believed that these efforts will increase the dissemination, implementation, and sustainment potential of programs in real-world settings. Practical tools exist for helping researchers to plan D4DIS and engage stakeholders in these efforts (see, for example, [35] and https://dicemethods.com/tool, accessed on 13 October 2021).

In summary, D&I science approaches can be used to systematically address the research-to-practice lag by actively engaging stakeholders in the design of interventions that fit the changing context and needs of end users—such as the economic climate when implementing a TWH program [36]—and examining the processes by which these interventions are adopted, implemented, and sustained in workplaces [30].

### Translational Science vs. D&I Science: Where Do They Overlap?

The proliferation of terminology to describe D&I activities has been explored extensively in the literature [37]. In the international OSH field, the terms knowledge translation, knowledge transfer, and knowledge transfer and exchange have been used [19,37,38,39,40,41] to describe similar or synonomous processes. At NIOSH [20,42], and in the context of TWH [11], the term translation(al) research is used, and while overlapping with the term D&I, it has important differences, as described below.

Translational science has been defined as, “the field of investigation which seeks to understand the scientific and operational principles underlying each step of the translational process” [43] (p. 456). As Fort et al. [44] state succinctly, translational research takes scientific discoveries “from the bench to the bedside and back again”. Or perhaps more appropriately for OSH, from the lab to the field (i.e., the worksite/workplace) and back again. Whereas traditional scientific inquiry is primarily concerned with creating new knowledge, translational science is ultimately focused on the process of applying existing evidence to address health-related problems to generate generalizable knowledge [43,45].

Translational science has been conceptualized as crossing all translational or “T” phases of the research continuum, from scientific discovery (T0), to efficacy (T1), effectiveness (T2), D&I (T3), and the outcomes and effectiveness of research in populations (T4) [44,46]. However, in practice, translational science has largely focused on barriers to intervention development at the efficacy and effectiveness stages (T1 and T2), while D&I science has focused on barriers to intervention adoption, use and sustainment (T3 and T4) [45]. More and better integration of D&I science across the translational continuum has therefore been called for [45,47].

Increasingly, OSH researchers in the United States are adopting the terminology of mainstream D&I science [19,42]. More work is needed to harmonize terminology so that research in this area can be characterized and synthesized to enhance the impact and understanding of it.

The following sections use the Curran [15] tool to expand on important concepts in the D&I field for TWH researchers to consider when planning/conducting D&I studies.

## 3. D&I in Plain Language

### 3.1. Does “the Thing” Work? Efficacy and Effectiveness Research

Research in D&I is related to, but distinct from, traditional efficacy and effectiveness research. Referring to the Curran definition [15], efficacy research evaluates the initial impact of an intervention (“the thing”) when it is delivered under optimal, controlled conditions. Effectiveness research looks at whether “the thing” works, determining the impact of an intervention with demonstrated efficacy to obtain more externally valid (generalizable) results [37]. As Anger and colleagues note in their systematic review of TWH interventions [3], the first step in identifying programs to disseminate and implement is to establish their effectiveness. As stated previously, while efficacy and effectiveness research are concerned with investigating specific interventions and health or safety outcomes in either ideal or real-world settings, D&I research is particularly concerned with the adoption, successful implementation, and sustainability of the intervention [48]. However, it is important to mention the integration study designs at the intersection of effectiveness and implementation research. These are referred to as hybrid effectiveness–implementation designs [49]. They exist on a spectrum with sub-types depending on the relative emphasis on effectiveness and/or implementation, with a primary focus on effectiveness regarded as type 1, equal attention to effectiveness and implementation referred to as type 2, and primary emphasis on implementation regarded as type 3 [49,50,51]. These hybrid designs relate to the aforementioned “designing for dissemination and sustainability” [34] and better integration of D&I science across the translational continuum [45,47]. This integration is important because if efficacy and effectiveness studies focus only on achieving the maximum effect, this will likely and unintentionally limit their application in real-world settings due to issues of cost, burden, poor fit with local context, lack of buy-in and available expertise for program implementation.

### 3.2. How Best to Do “the thing”? A Very Brief Overview of D&I Models, Theories, Frameworks, Methods, and Measures

Once the “thing” (i.e., the intervention) is developed and tested, the focus of D&I efforts is on “doing the thing” well, which entails the selection of an appropriate theory, model, or framework (TMF) to guide the research. While the terms “theory”, “model”, and “framework” have distinct meanings, they are often used interchangeably [52]. TMFs generally describe tools to plan, evaluate, or understand barriers and facilitators (known as determinants) to D&I processes [16,53,54]. These tools help researchers on the front-end to plan, organize, and understand D&I phenomena, and on the back-end to understand why/how D&I strategies succeed or fail [24]. Table 2 highlights select D&I TMFs such as the Consolidated Framework for Implementation Research (CFIR) [55], the RE-AIM (Reach, Effectiveness, Adoption, Implementation, Maintenance) framework [8,9], the EPIS (Exploration, Planning, Implementation, Sustainment) framework [56], the diffusion of innovations theory [31], and the Theoretical Domains Framework (TDF) [57]. The CDC’s Knowledge to Action Framework [58] is another example of a tool developed for use by public health researchers to explore how evidence-based interventions can be translated into effective programs, policies, and practices. More than 150 D&I TMFs have been identified in the literature [21,22,23], and TWH researchers may wonder how to select an appropriate one. Fortunately, several efforts have focused on collecting and synthesizing the proliferation of TMFs available for D&I research. A key, publicly available resource, the D&I Models in Health (Table 2), provides an interactive webtool for study planning, combining and adapting TMFs, and selecting measurement tools to assess important D&I constructs [24].

While there have been a large number of D&I TMFs proposed, leading some scholars to perceive the field as a “Tower of Babel” [65], there are more commonalities than differences across TMFs on factors known to affect the adoption, implementation, and sustainment of evidence-based interventions [66]. Key constructs commonly considered include multilevel contexts (i.e., inner and outer contexts with multiple layers within each, as described above) [8,9,56,61], characteristics of recipients at multiple levels [9,55], intervention and implementation strategy characteristics, and considerations for the various stages or phases of D&I (e.g., exploration, preparation, reach, adoption, implementation, sustainment, and important outcomes) [8,9,56,61].

An example of the use of D&I models in OSH is from Tinc et al. [67], who employed the CFIR [55] to evaluate, among key stakeholders, the success of implementing a national program to prevent tractor rollover deaths in the United States with the use of a rollover protection structure (ROPS). The study used stakeholder surveys to assess short- and long-term outcome measures (intakes, funding progress, and tractor retrofits) and identify which CFIR components correlated with these outcomes. Results indicated that eight CFIR survey items reflecting four constructs—access to knowledge and information, leadership engagement, engaging (in fundraising and funding requests), and reflecting and evaluating—were highly correlated with at least one of the outcomes. In the TWH literature, Nobgrea and colleagues [36] used the RE-AIM framework [8,9] to iteratively develop and evaluate a toolkit to enable workplace safety and health practitioners to implement their own participatory TWH programs. An example from Europe [68] involves a process of using a D&I framework to systematically assess adaptations to an occupational health intervention. While these studies describe promising applications of D&I in OSH/TWH, more work is needed to generate generalizable knowledge about “how best to do the thing” in diverse types of workplaces with multilevel stakeholders, such as workers, employers and supervisors, union representatives, and regulators/policy makers.

Another critical aspect of “doing the thing” is selecting the correct methods, designs, and measures. D&I science methods are varied, include both qualitative and quantitative techniques [25,26], and increasingly focus on the use of pragmatic, participatory, and mixed-method approaches [69]. Hybrid effectiveness–implementation designs [49,50,51] mentioned previously, promote the examination of both effectiveness and implementation outcomes within the same study to speed-up the research-to-practice process. Brown and colleagues [25] compiled a useful compendium of other D&I study designs. These include within-site designs to evaluate the success of intervention implementation inside a workplace or community, between-site designs that compare implementation processes among sites having different exposure conditions, within- and between-site comparisons with rollout designs where intervention start times are staggered, and factorial designs to examine multiple implementation strategies.

Examples of D&I measures that can be used for “doing the thing” that are reliable, have validity data, and are considered to be pragmatic [28] are presented in Table 2, as are resources for identifying commonly used D&I tools, instruments and measures. In the D&I field, using previously developed measures is encouraged so that intervention findings may be compared across studies [64]. While it is not possible to characterize the full extent of available and appropriate D&I methods, designs, and measures, the above-mentioned resources are a starting point for TWH investigators to learn more about what considerations are needed when conducting rigorous D&I studies. It is also important to note more recent calls to integrate a health equity perspective across and within D&I TMFs, methods, and measures, whether health equity is a central focus of the D&I study or not [70].

### 3.3. D&I Strategies: The “Stuff” Researchers Do to Help Others Do “the Thing”

Referring again to the Curran’s [15] plain language explanation, implementation strategies are “the stuff” researchers do to try to help people (e.g., workers and employers)/(work)places “do the thing” as designed/intended. Dissemination and implementation strategies are a collection of methods or techniques to enhance the adoption, implementation, and sustainability of an evidence-based intervention [71,72]. In other words, D&I strategies are activities, approaches, and processes used to spread/deliver interventions to target populations and/or integrate interventions in target settings [73,74,75]. Key strategies have been classified in the Expert Recommendations for Implementing Change (ERIC) compilation, which identified 73 distinct strategies across nine domains: (1) use evaluative and iterative strategies; (2) provide interactive assistance; (3) adapt and tailor context; (4) develop stakeholder interrelationships; (5) train and educate stakeholders; (6) support clinicians; (7) engage consumers; (8) utilize financial strategies; and (9) change infrastructure [76]. The ERIC strategies have been adapted for school settings [77] and may be modified for use in workplaces. Determining which implementation strategies (or bundles of strategies) are the most appropriate is context dependent. Research to identify effective implementation strategies to “do the thing” within workplace settings is nascent [78], as are efforts to capture implementation outcomes, as is described in the next section.

### 3.4. D&I Outcomes: How Much and How Well They “Do the Thing”

Implementation outcomes reflect how much and how well intervention implementers “do the thing” [15]. It is important to note that implementation outcomes, described in Table 3, are distinct from multilevel effectiveness outcomes which are often assessed in TWH studies. These effectiveness outcomes may include well-being, physical and mental health, occupational injuries, illness and fatalities, work-related fatigue, work stress, job performance, job satisfaction, safety climate, work–life balance [3] and occupational health equity [79].

As described previously, studies in D&I are typically conducted after program efficacy and effectiveness are demonstrated and an evidence base has been established. Building on the effectiveness evidence base, D&I science outcomes focus on for whom and in what contexts the intervention works [14,80]. A focus on health equity and pragmatic research methods and measures is also important [25,27,28], as discussed previously in this paper. Furthermore, research on mechanisms of change/action that describe the process by which implementation strategies bring about specified implementation outcomes (i.e., mediational analyses) is recommended [81]. Without understanding how implementation strategies work, they will likely fail to achieve a positive impact [82].

### 3.5. Context Matters

As stated, although not included in the Curran [15] conceptualization, another key feature of D&I is the importance of context, which has been defined as the unique circumstances within which intervention implementation is embedded [83]. Context is dynamic, multilevel, and cuts across economic, political, social, and temporal domains [17,84,85]. Factors at the system, organizational, worksite, and individual levels can serve as facilitators or barriers to implementation [85]. Characteristics of the intervention itself [31,86], as well as the intervention–context fit, can also have an impact on implementation outcomes, and all of these contextual influences may be present/active at different stages of the implementation process [75].

Despite its importance, context is one of the least often reported elements in research [17,85]. A recent scoping review of 17 determinant frameworks in implementation science indicates that most frameworks provide only a limited description and definition of context, and there is inconsistency with regard to which contextual determinants are addressed [16]. As May and colleagues [87] note, context “is a problem” as many efficacy/effectiveness studies and even some D&I research designs try to “control out” contextual confounders, even though these represent the real-world conditions into which interventions must be integrated. Traditional randomized controlled trials do not typically answer the question about why or how intervention impact varies by setting, focusing instead on questions related to internal validity [33]. Despite the challenges and tensions noted, gaining an understanding of context is critical for determining program/policy outcomes [33], including in TWH studies.

While researchers have demonstrated the value of considering multilevel contexts and stakeholder engagement in designing TWH interventions [2,11,36,88], a systematic review of TWH studies by Feltner and colleagues [18] identified only a limited number of interventions focusing on multilevel contextual factors, such as work organization and union membership status, health insurance status, access to primary care services, management support, availability of resources, and employee stress or strain related to company downsizing. No studies were identified that systematically assessed possible variation in intervention effectiveness by individual, worksite, organizational, or community factors [10]. Given the variety of contexts in which the TWH model can be implemented and studied—with variations in employers, work environments, and workers—understanding the factors that influence the effectiveness of integrated interventions is important, and more research is needed in this area [10].

Moreover, it has been suggested that TWH studies should include a focus on complex systems approaches [89] to gain a deeper understanding of the multilevel influences within the TWH framework acting on intervention outcomes. Understanding context as a “process” rather than a “place” (i.e., the physical environment in which a practice is embedded) acknowledges that the setting in which implementation occurs is the product of “continuous accomplishments” and requires constant negotiation and iteration [87] (p. 4). D&I approaches can help address some of the complex, non-linear systems issues that impact TWH and OSH more generally.

### 3.6. Putting It All Together: A Logic Model for D&I in TWH

**Figure 1:**
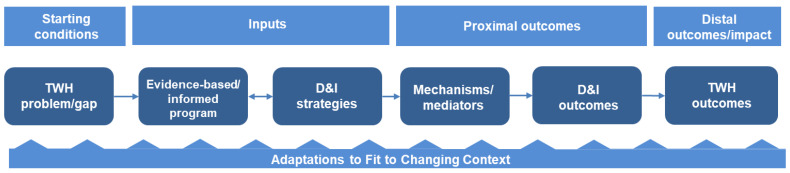
Logic model of a D&I study for TWH. Source: adapted from [91].

To illustrate the D&I process for TWH as described throughout this paper, a logic model is presented in Figure 1. Referring to the model, D&I outcomes (and ultimately work-related health, safety, and well-being outcomes) are conceptualized to be influenced by (a) the intervention (the evidence-based/informed program), (b) the D&I strategies, (c) mechanisms/mediators, or the “how and why” an implementation strategy operates [81], and (d) adaptations to context [9,90], such as modifications that will need to be made iteratively to sustain the initiative over time (e.g., lower cost, different staff/expertise, staff attitudes/buy-in, and different settings). The logic model displays both proximal outcomes, such as adoption and fidelity [37,48], and more distal outcomes and impacts, such as reduced occupational morbidity and mortality, enhanced well-being, and occupational health equity.

### 3.7. A Few Other Important D&I Distinctions for TWH Researchers

Despite calls for increasing the emphasis of D&I in TWH studies [3,11,18], the application of these approaches has been limited [10,18]. One reason may be due to a lack of clarity among TWH researchers on what D&I science is—and is not. Recent papers for the broader D&I community elaborate on this issue, addressing misconceptions about specific D&I frameworks [92]. The following section presents a few concepts potentially requring clarification for the TWH context, as garnered from the literature.

**D&I is not r2p (but there is overlap).** At NIOSH, r2p (research to practice) is an approach to communicate and transfer NIOSH “knowledge, interventions, or technologies” to relevant stakeholders for use in workplaces to contribute to reducing and eliminating injuries, illness, and fatalities [93]. In short, the NIOSH r2p program focuses on the transfer of interventions into effective practice. In contrast, D&I—or part of what NIOSH currently refers to as translation research—is the systematic study of these efforts [19,94]. While there are areas of overlap, including the focus on engaging stakeholders, r2p and translation research should be considered as separate, albeit complementary, areas of focus and effort.

**D&I is not the same as program evaluation (but there is overlap).** CDC defines program evaluation as the systematic collection of information on the activities, characteristics, and results of programs in a specific setting to inform local knowledge and practice [95]. Examples of program evaluation may include formative, process, or summative activities [96]. While the boundaries are unclear, an important distinction can be made between the science of improvement (program evaluation) versus the science of dissemination and implementation [96]. Whereas program evaluation might consider intervention outcomes (and even in different settings), the focus is on how to make the intervention itself (i.e., “the thing”) better. In contrast, D&I outcomes relate to how to integrate the intervention into a variety of settings with a focus on methods and measures and the impact of implementation strategies [25,80]. For example, whereas program evaluation may focus on organizational and behavioral outcomes of a worksite injury prevention program, D&I is focused on the outcomes of acceptability, integration, and sustainability of the specific program/policy/practice [9,37,48] in different workplaces and community settings.

In summary, D&I science is concerned with dynamic, multilevel contextual factors related to the characteristics of the intervention, implementation strategies, individual, program provider, organizational (workplace), and policy/regulatory levels; pragmatism; and sustainability [47,86]. D&I processes should be considered as a set of complex, non-linear, and iterative accomplishments that are emergent and dynamic [87]. Given this multilevel and systems-level approach, D&I is well-aligned with the OSH field. As noted previously, D&I (also referred to as translation(al) research) has been integrated into strategic NIOSH initiatives [19,20,42]. The case study presented below provides an extended example of identifying an evidence-based, OSH program, adapting it for TWH, and moving it along the translational research continuum [20,97,98,99,100]. Such work sets the stage for future opportunities for D&I science.

## 4. Translating an Evidence-Based, Young Worker Program for TWH: A Case Study Example

In the United States, young workers (aged 15–24 years) experience higher rates of job-related injury than adult workers (aged 25–44 years) [101]. During 2012–2018, an estimated 3.2 million nonfatal injuries to young workers were treated in hospital emergency departments [101]. Work-related injuries may be life-altering, and young people hurt at work may experience a “cumulative burden of morbidity” over their lifetimes [102]. Data from the U.S. Bureau of Labor Statistics, Census of Fatal Occupational Injuries, indicate that 2349 young workers died on the job during the 2011 through to 2017 period [103]. Although employers are required by law to provide basic safety training to all workers, the lack of quality safety training has been shown to be a contributor to occupational injury among young workers [104,105].

To address this public health problem, NIOSH and its partners developed the classroom-based, “Youth @ Work: Talking Safety” curriculum [106,107,108] to provide youth with a foundation of OSH competencies before they enter the workforce [109]. Results from quasi-experimental and (on-going) randomized trials indicate the effectiveness of the NIOSH curriculum to have a positive impact on adolescent students’ work safety knowledge, attitudes, norms, self-efficacy, and intention to enact safety behaviors in the workplace [110,111].

Building on the evidence base for the “Youth @ Work: Talking Safety” curriculum, Promoting U through Safety and Health (PUSH), an online training tool for young workers, was developed by researchers with the Oregon Healthy Workforce Center, a NIOSH Total Worker Health Center of Excellence [112]. PUSH expands “Talking Safety” to include TWH concepts in an online format (versus the classroom format of “Talking Safety”) and for delivery in a different context (city park departments versus middle schools and high schools). The adaptation was based on inputs gathered through a needs assessment conducted with young people aged 14–24 years employed as aquatics workers in a city parks and recreation program in Portland, Oregon [112,113,114]. This work allowed the project team to qualitatively assess the acceptability and fit of the proposed OSH and health promotion content for the target population and establish the feasibility of an online delivery model [112,113]. To assess intervention outcomes, an individual-level randomized controlled trial was conducted with 140 young aquatics workers [114]. Most intervention workers (compared to the control group) reported learning new information (95%), liking the training (59%), and indicated that they had adopted new healthy behaviors (63%). Furthermore, the parks program indicated that the online format was practical and easy to administer.

In a subsequent project phase, the PUSH training was scaled out (or disseminated in D&I terms) to young workers in a range of occupations, including cashiers, accountants, service managers, counselors, and lifeguards [115].

Referring to the translational research pipeline described earlier in this paper [43,44,45,46,97,98,99,100], the “Youth @ Work: Talking Safety” curriculum was developed and tested in T0 and T1, with further effectiveness testing conducted in T2. The evidence base established for the curriculum was leveraged to develop PUSH with a TWH focus and tested it with other target groups in different contexts, focusing largely on barriers to intervention development at the efficacy and effectiveness stages (T1 and T2). Future D&I studies (T3) involving PUSH could, for example, focus on the testing of theories, models, and frameworks for D&I processes, some of which have been described in this article [21,22,23,24]. Research could be conducted to systematically identify, develop, test, evaluate, and/or refine strategies [73,76] to disseminate and implement PUSH interventions in new community/workplace contexts where young workers are employed. Another example of future opportunities for D&I research would include a systematic investigation of the local adaptations [90,116,117] of PUSH in the context of its implementation within various settings that employ young workers. Studies of influences on the development, packaging, transmission, and reception [118] of PUSH in various settings and contexts where young workers can be reached would also help to move this promising TWH research into (sustained) practice.

## 5. Discussion

The aim of D&I is to gain an understanding of the contextual factors—including the needs and priorities of stakeholders at each level [119]—that facilitate and hinder the successful uptake of evidence-based interventions. Although D&I science holds promise for speeding up the translation of TWH interventions to enhance the health and well-being of the global workforce, research in this area remains limited. One barrier to the widespread uptake of D&I may be the need to identify interventions that are ready for translation. As Anger and colleagues [3] note, “Perhaps it is premature to press for dissemination of the TWH programs until their effectiveness is better established.” (p. 243). However, waiting for interventions to meet evidence standards may contribute to the translational lag time while also generating interventions that are not replicable in the real world [84,120]. Types of evidence more typical in OSH than that generated through highly controlled trials include guidelines, recommendations, and observational as well as worker case studies [121]. Communicating which TWH initiatives are promising (i.e., evidence-informed versus evidence-based [37,122]) may speed-up practical and empirical outcomes.

To ensure that TWH interventions are replicable in real-world settings, efficacy and effectiveness studies should be designed and conducted with an eye toward feasibility, generalization, dissemination, equity, and sustainability at every phase of the research continuum [9,45]. The concept of designing for dissemination, implementation, and sustainment (D4DIS), mentioned previously, is a process that can be used to ensure that the products of research are developed with the needs, resources, and time frames of the target audience in mind [34]. Practical tools exist to help researchers plan D4DIS and engage stakeholders in these efforts (see, for example, DICEmethods.org). Moreover, D&I science TMFs such as RE-AIM [8,9,92], CFIR [55], and EPIS [56,61] can be used not only for evaluating interventions but can also guide their planning with stakeholders (e.g., workers, employers, and community members), be used iteratively during implementation to adapt the program/practice to better fit the context (e.g., local workplaces), and respond to emerging implementation data. More and better integration of D&I across the translational science continuum has been called for to move research more rapidly into practice to benefit the public [45,47], including workers [42]. Moreover, by integrating D&I early in the research process, activities related to getting scientific innovations into the hands of end users may not be viewed as a burdensome, add-on activity [42], as someone else’s role [34,123,124], or as up to the stakeholders to figure out [125].

Consistency in how terminology is applied will also help to facilitate the uptake of D&I science in TWH and to develop generalizable knowledge about D&I outcomes. Rabin and colleagues [37] have assembled extensive glossaries to capture and harmonize the D&I “Tower of Babel” [65]. A project being conducted under the NIOSH Evaluation Capacity-Building initiative [126] is to develop, with D&I science scholars and internal and external stakeholders, a glossary of D&I research terms for OSH researchers. As with any developing field, there are minor differences in terminology and a relative emphasis on different theories, models and frameworks. However, within D&I, there is general agreement on key principles, factors, and methods (see Table 1; also [66]).

Another challenge may be limited D&I expertise in the TWH field, as is the case more generally [127]. Furthermore, although the broader D&I science field has expanded and diversified over the years, more investment in resources tailored to meet the needs of OSH and TWH researchers are required to build capacity with D&I science models, theories, frameworks [21,22,23,24], methods and designs [25,26], and pragmatic measures [27,28]. These tools can be used to conduct rigorous studies that bridge the gap between the lab, the field, and public health impact.

Another important area of focus for D&I in TWH is how to critically infuse an equity approach into all research endeavors [79,128,129,130]. This includes focusing on equitable reach from the beginning of the intervention planning and development; designing and selecting programs for populations at disproportionate risk for work-related injury, illness and reduced well-being; implementing what works; and developing implementation strategies that can help reduce inequities in OSH [129]. Practical issues—such as the complexity of accessing many workplaces and workers, especially those in smaller and low-resource businesses and who may experience multiple OSH inequities [129,131]—make conducting D&I research challenging [42]. However, D&I research in TWH can help to build the necessary evidence base for employers to adopt a more holistic, feasible, and sustainable approach to promote (current and future) employee health and well-being.

Finally, D&I approaches that consider multilevel contextual factors in TWH that influence intervention outcomes [18] are needed to address the dynamic, complex and emergent nature of twenty-first-century challenges in OSH [6,7,90], including global pandemics. TWH research is needed that systematically considers the factors at the system, organizational, worksite, and individual level that serve as facilitators or obstacles to implementation [31,75]. Designing implementation strategies to address contextual barriers [75] may help TWH researchers to consider multilevel influences (such as regulatory changes, or individual worker acceptance of new technologies) as well as other complex phenomena that influence the effectiveness, equity, adoption, implementation and sustainability of TWH programs, both in the United States and internationally [130].

## 6. Conclusions

Applications of D&I hold promise for addressing the limited movement of intergrated worker protection and health promotion interventions into widespread and sustained practice. Several reasons, including the need to identify promising TWH interventions that are ready to be moved along the research to practice continuum and limited D&I capacity, have been identified that may be potential barriers to the uptake of D&I approaches. However, numerous opportunities also exist. This paper drew upon a synthesis of the D&I science literature to provide TWH researchers and practitioners with an overview of the D&I field and a plain language explanation of key concepts. This analysis also presents D&I examples and resources, and discusses how D&I tools can be used to more rapidly deploy effective TWH programs. The end goal is to improve the current and future safety, health and well-being of working people and enhance occupational health equity in an increasingly dynamic and complex global economy.

## Figures and Tables

**Table 1:** Considerations for the use of D&I science approaches for rigorous, rapid, and relevant TWH initiatives (adapted from Glasgow and Chambers [12]).

Characteristics	Implications	TWH Considerations
Systems Perspective		
Context is critical	Research should focus on and describe context	What are the key circumstances and factors within and outside of the workplace that influence the uptake, implementation, and sustained use of TWH programs?
Multilevel complexity needs to be considered	Most problems and interventions are dynamic, multilevel, and complex	What levels of influence (policy/regulatory, community, organizational, managers/supervisors, worksite/team, and individual workers) are addressed?
Focus on systems characteristics	More emphasis needed on interrelationships among system elements and system rules	Were there specific resources, values, or missions that drove the success or hindrance of the TWH effort? Are there unintended consequences?
**Robust, Practical Goals**		
Representativeness and reach	Focus on reaching broader segments of the population and those most in need	Of the eligible workers, who participated? What recruitment efforts could be made for more equity and inclusion?
Generalizability	Study generalization and replication (or lack of such) across workplace settings	What efforts are made to ensure the TWH intervention can be scaled to other settings and delivered by other staff?
Pragmatic and practical	Producing answers to specific questions relevant to stakeholders at reasonable costs	What needs assessments were conducted? How is the fit of the TWH effort with stakeholder needs measured/assessed? Are the costs realistic/feasible?
Scalability and sustainability	From the outset, a greater focus on scale-up potential and likelihood of sustainability (designing for dissemination and sustainment [34])	What are the startup and sustainability resources and costs the setting will need to consider for full institutionalization? What adaptations will need to be made (iteratively) to support continued use of the TWH program and scale-up to other setting/systems?
**Research Methods to Enhance Relevance**		
Rigorous	Identifying and addressing plausible threats to validity in context of questions and a greater focus on replication	What is the best research design to answer the specific TWH question? What are the details necessary for replication?
Rapid	Approaches that produce faster answers	What is the current translational lag time? In what ways are research efforts speeding up that process?
Adaptive	The best solutions usually evolve over time, as a result of informed hypotheses and iterative mini-tests with feedback	What are the core elements of the TWH effort (things that cannot change) and what things need iterative assessment? How are these core components communicated to those external to the team? How much guidance is provided for adaptation to local context?
Integration of methods; triangulation	For greater understanding, integrated quantitative and qualitative methods are often required	Who and what experiences in the TWH effort need to be captured to understand the richer context and outcomes? What are the best methods for this?
Relevant	Relevance to stakeholders should be a top priority	Do stakeholders find the TWH effort a high priority, feasible, acceptable, and appropriate? What are strategic ways to obtain unbiased responses?
Equitable	All stakeholders and relevant community sectors are represented and engaged	Does the intervention advance/support occupational health equity? Are hard-to-reach and hardly reached worker groups engaged? Are program outcomes equitable? Can the intervention be conducted in settings frequented by and serving disadvantaged populations? Does it unintentionally enhance OSH disparities?
Respect for diverse approaches; humility	Different perspectives, goals, methods and approaches are needed. Continuing the same existing approaches will produce the same unsatisfactory results	What about the research is novel, comprehensive, and holistic? How is this communicated to various stakeholders? Are multiple disciplines involved?

**Table 2:** Select D&I theories, models, frameworks, tools, and resources.

Tool/Resource	Brief Description
Select D&I Theories, Models, and Frameworks (TMFs)	
Consolidated Framework for Implementation Research (CFIR) [55]	A widely used D&I framework that considers a range of implementation determinants (i.e., barriers and facilitators). The CFIR comprises five major domains (the intervention, inner and outer settings, the individuals involved, and the implementation process). Within these domains, multiple constructs reflect determinants of implementation. For example, complexity and cost are constructs within the intervention characteristics domain; external policies are a construct in the outer setting domain; culture is a construct within the inner setting domain; planning and engaging are constructs within the process domain; and the characteristics of the individuals involved domain focuses on individual-level constructs such as self-efficacy and knowledge, attitudes, and beliefs about the intervention [55].
RE-AIM (Reach, Effectiveness, Adoption, Implementation, Maintenance) framework [8,9]	A widely used D&I framework that includes five key dimensions: Adoption, Reach, Implementation, Effectiveness, and Maintenance. RE-AIM was designed to enhance the quality, efficiency, and public health impact of interventions. Cutting across all five of the RE-AIM implementation outcomes are equity concerns related to the representativeness of those who participate or benefit from evidence-based programs. The RE-AIM framework can be used for intervention planning, evaluation, and (iteratively) guiding adaptations to implementation strategies.
Practical, Robust, Implementation, and Sustainability Model (PRISM) [9,59,60].	An extension of the RE-AIM framework, PRISM considers key contextual factors that influence implementation at multiple socioecological levels. PRISM contextual factors include: the program characteristics from the perspective of organizational and individual recipients, the characteristics of diverse, multilevel recipients of the program, the implementation and sustainability infrastructure, and the external environment. PRISM may be used to guide researchers during the program planning, implementation, evaluation, and dissemination phases.
EPIS (Exploration, Planning, Implementation, Sustainment) framework [56,61]	The EPIS framework consists of four phases (Exploration, Planning, Implementation, Sustainment) that align with the implementation process, the identification of outer system and inner organizational contexts, and “bridging and innovation factors” that are concerned with the intervention being implemented and the interaction between outer and inner contexts.
Diffusion of innovations theory [31]	A widely used theory that seeks to explain the processes and factors influencing the spread and adoption of innovations through certain channels over time, considering components such as perceived characteristics of the innovation; innovativeness of the adopter; social system(s); individual adoption processes; and the diffusion system.
Theoretical Domains Framework (TDF) [57]	A compilation of theories relevant to implementation that resulted from a systematic review of published D&I frameworks.
CDC Knowledge to Action (K2A) Framework for Public Health [58]	A framework that can be used to explore how evidence-based interventions are translated into effective (public health) programs and practices. The framework consists of three phases: research, translation, and institutionalization. Under each of the three phases are supporting structures and evaluation.
D&I Models in Health: www.dissemination-implementation.org (accessed on 13 October 2021)	A free interactive webtool for the selection of TMFs that can be used for study planning, combining and adapting TMFs, and selecting measurement tools to explore D&I constructs.
***Examples/Select D&I Measures, Instruments, and Tools***	
Acceptability of Intervention Measure (AIM), Intervention Appropriateness Measure (IAM), and Feasibility of Intervention Measure (FIM)	Measures by Weiner and colleagues [62] to assess intervention acceptability, appropriateness and feasibility (12 items, four for each construct).
The Program Sustainability Assessment Tool (PSAT): https://sustaintool.org/psat/	A reliable, 40-item instrument from Luke and colleagues [63] with eight domains (5 items per domain) that can be used to assess the capacity for the sustainability of public health programs.
The EPIS framework website: https://episframework.com/measures (accessed on 13 October 2021)	Provides examples of and free access to webinars and other quantitative measures that assess constructs for inner (such as individual program adopter characteristics) and outer (such as sociopolitical and economic contexts that influence the process of implementation) contexts [56,61].
The Society for Implementation Research Collaboration Instrument Review Project: https://societyforimplementationresearchcollaboration.org/sirc-instrument-project (accessed on 13 October 2021)	Provides an overview of measures for both implementation outcomes and multilevel contextual domains. This resource requires a membership for access.
The National Cancer Institute, Grid-Enabled Measures Database (GEM): https://www.gem-measures.org/Public/Home.aspx (accessed on 13 October 2021)	Houses tools to enhance the quality and harmonization of measures for D&I [64].
The CFIR website: cfirguide.org (accessed on 13 October 2021)	Provides free access to data collection tools based on CFIR constructs [55].
The RE-AIM and PRISM frameworks website: https://www.re-aim.org (accessed on 13 October 2021)	Provides free templates of focus group and one-on-one interview guides for assessing RE-AIM constructs [8,9] before, during, and after program implementation. The website also includes videos, interactive tools, and presentations on PRISM/RE-AIM.

**Table 3:** Examples of implementation outcomes adapted for TWH interventions.

**Acceptability**	Perception among key stakeholders that the TWH program or practice is agreeable or satisfactory
**Adoption**	Agreement among key stakeholders and settings to use a TWH intervention (i.e., “uptake”)
**Appropriateness**	Perceived fit of the TWH intervention for a given context/population/health and safety problem
**Costs**	Resources needed for the uptake, implementation, and sustainment of TWH interventions
**Feasibility**	Extent to which the TWH intervention can be used successfully within a given workplace setting
**Fidelity**	Degree to which a TWH intervention is implemented as intended by program developers
**Penetration**	Extent of integration of a TWH intervention within a workplace, community, or system
**Reach**	Absolute number, proportion, and representativeness of individuals who are willing to participate in a given TWH initiative, intervention, or program, and reasons why or why not
**Adaptation**	Degree to which evidence-based TWH initiatives are modified to better fit with the local context
**Sustainability**	Extent to which a newly implemented TWH intervention is maintained or institutionalized within an organization/workplace

Sources: Adapted from Glasgow et al. and Proctor et al. [9,48]. For more details and comprehensive definitions see Rabin and Brownson [37].

## Data Availability

Not applicable.
